# Supplementary material for: CT-Detected Subsolid Nodules: A Predictor of Lung Cancer Development at Another Location?
Source: Cancers (Basel). 2021 Jun 4;13(11):2812. doi: 10.3390/cancers13112812 (PMC8200192; doi:10.3390/cancers13112812)
Supplement: Supplementary file 1 [file cancers-13-02812-s001.zip › cancers-1225266-supplementary.pdf]

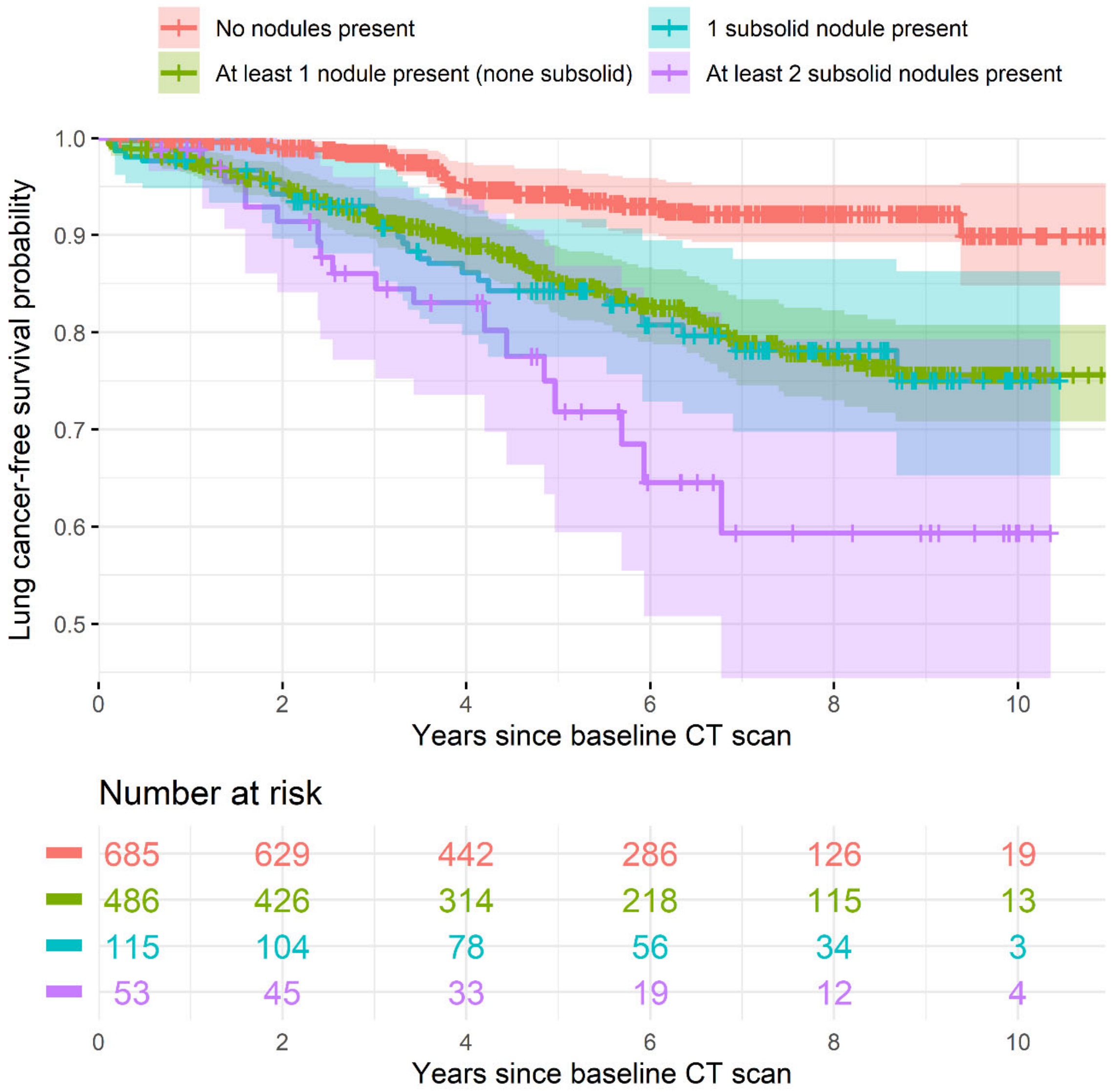

Figure S1: Lung cancer probability by nodule type present at baseline (with confidence intervals). This figure shows a Kaplan-Meier curve of lung cancer-free survival, including a "number at risk" table. Vertical dashes on a line indicate the end of patients' follow-up. 95% confidence intervals are given as transparent fills of the same color.

Table S1: Univariable Cox regression for lung cancer risk prediction.

**Variable**	**Beta coefficient**	**Hazard ratio (95% confidence interval)**	***p* value**
**Patient characteristics**			
Age at earliest scan, per year $(((x - 39) / 10)^2)$	0.7044	2.02 (1.61 to 2.54)	<0.001
Age at earliest scan, per year $(((x - 39) / 10)^3)$	−0.1671	0.85 (0.80 to 0.89)	<0.001
Male sex	0.1217	1.13 (0.80 to 1.59)	0.48
**CT features excluding nodule type**			
Emphysema	1.1874	3.28 (2.34 to 4.60)	<0.001
Bronchitis	0.8794	2.41 (1.71 to 3.40)	<0.001
Interstitial lung disease	4.4566	4.46 (1.96 to 10.14)	<0.001
Lymphadenopathy	0.7423	2.10 (1.44 to 3.06)	<0.001
Aortic calcifications	0.9580	2.61 (1.84 to 3.69)	<0.001
Coronary calcifications	0.8599	2.36 (1.65 to 3.39)	<0.001
Nodule present	1.6123	5.01 (3.33 to 7.56)	<0.001
Mean diameter of largest nodule, per mm	0.03449	1.04 (1.02 to 1.05)	<0.001
**Nodule type information**			

Largest nodule is a SSN	1.0433	2.84 (1.85 to 4.37)	<0.001
Solid nodule present	1.2698	3.56 (2.51 to 5.05)	<0.001
Solid nodule count, per nodule	3.0830	21.82 (9.07 to 52.53)	<0.001
Solid nodule count, per nodule ($(x + 1) \times \ln(x + 1)$)	−1.3605	0.26 (0.17 to 0.40)	<0.001
Part-solid nodule present	1.0499	2.86 (1.59 to 5.13)	<0.001
Part-solid nodule count, per nodule	0.2717	1.31 (1.03 to 1.67)	0.026
Ground glass nodule present	0.9238	2.52 (1.60 to 3.96)	<0.001
Ground glass nodule count, per nodule	0.4608	1.59 (1.25 to 2.02)	<0.001
Perifissural nodule present	−0.2966	0.74 (0.43 to 1.29)	0.29
Perifissural nodule count, per nodule [1]	−0.1796	0.84 (0.58 to 1.21)	0.34

Dashed lines between two rows indicates that both transformations of the variable were included in the regression analysis to obtain the given hazard ratios and p values. Some variables were transformed as indicated by the mathematical expression in brackets, in which x represents the untransformed variable. N/A, not applicable.

Table S2: Parsimonious lung cancer risk model excluding nodule type information.

**Variable**	**Beta coefficient**	**Hazard ratio (95% confidence interval)**	***p* value**
**Patient characteristics**			
Age at earliest scan, per year $(((x-39)/10)^2)$	0.52318	1.69 (1.30 to 2.19)	<0.001
Age at earliest scan, per year $(((x-39)/10)^3)$	−0.13967	0.87 (0.82 to 0.92)	<0.001
Male sex	−0.27946	0.76 (0.50 to 1.14)	0.18
**CT features excluding nodule type**			
Emphysema	0.77266	2.17 (1.44 to 3.26)	<0.001
Bronchitis	0.39805	1.49 (0.99 to 2.23)	0.055
Interstitial lung disease	0.91210	2.49 (0.94 to 6.59)	0.066
Lymphadeno-pathy	0.46593	1.59 (1.01 to 2.51)	0.045
Aortic calcifications	0.45241	1.57 (1.01 to 2.46)	0.047
Coronary calcifications	0.49004	1.63 (1.04 to 2.56)	0.033
Nodule present	1.36447	3.91 (2.51 to 6.11)	<0.001
Mean diameter of largest nodule, per mm	N/A	N/A	N/A

A parsimonious model was derived using Cox regression containing only variables with p value < 0.20 using backwards elimination. Some variables were transformed as indicated by the mathematical expression in brackets, in which x represents the untransformed variable. N/A, not applicable.

References

1. de Hoop B, van Ginneken B, Gietema H, Prokop M. Pulmonary Perifissural Nodules on CT Scans: Rapid Growth Is Not a Predictor of Malignancy. *Radiology*. **2012**; *265*, 611–616, doi:10.1148/radiol.12112351.
